# Saturation effect of anion gap in predicting long-term mortality in critically ill patients with chronic obstructive pulmonary disease (COPD): A retrospective cohort study based on the MIMIC-IV database

**DOI:** 10.1097/MD.0000000000041364

**Published:** 2026-04-17

**Authors:** Xiaohan Xiu, Zhenyu Yang, Xuemei Chen

**Affiliations:** a Department of Critical Care Medicine, The First Affiliated Hospital of Chongqing Medical University, Chongqing, China; b Heilongjiang University of Chinese Medicine, Harbin, China.

**Keywords:** anion gap, chronic obstructive pulmonary disease, MIMIC IV database

## Abstract

The relationship between anion gap (AG) and long-term mortality in intensive care unit has been widely reported, but whether this association exists in critically ill patients with chronic obstructive pulmonary disease (COPD) is still unknown. The data of this study were collected from the Medical Information Mart for Intensive Care-IV. First of all, we used the Cox regression analysis and Kaplan–Meier curves to measure the relationship between AG and 365-day mortality for critically ill patients with COPD. Next, a restricted cubic spline was used to analyze the relationship between AG and mortality. Finally, age, sex, weight, hypertension, type 2 diabetes mellitus, heart failure, myocardial infarction, and chronic kidney disease were considered for subgroup analysis. A total of 2594 eligible subjects were sampled, of which 36.24% died within 365 days of intensive care unit admission. Cox regression analysis, after adjusting for confounders, demonstrated a significant association between AG and 365-day mortality in patients with COPD (hazard ratio = 1.03, 95% confidence interval: 1.01–1.05. *P* < .05). Stratifying AG into quartiles revealed higher levels of AG associated with an increased risk of death (Q1: 1.00, Q2: 1.34 [1.09–1.66], Q3: 1.44 [1.17–1.78], and Q4: 1.49 [1.19–1.87]). Additionally, restricted cubic spline analysis indicated a nonlinear relationship, with a critical value of AG at 14 mmol/L. Subgroup analysis highlighted AG as a significant predictor of long-term mortality in COPD patients across different subgroups, with an interaction effect observed in the subgroup with type 2 diabetes mellitus. In critically ill patients with COPD, there was a significant positive association between AG and 365-day mortality. In addition, there is a saturation effect at AG of 14 mmol/L.

## 1. Introduction

Chronic obstructive pulmonary disease (COPD), a respiratory disease causing progressive airflow limitation and decline in lung function, is the most common disease. It is reported that the global prevalence was estimated to be 10.6% accounting for 480 million cases in 2020, which is expected to increase to a total of 592 million by 2050,^[1]^ and COPD remained one of leading causes of death worldwide with millions of death annually.^[2]^ Comorbidities are common among COPD patients, including cardiovascular disease, metabolic disorders, skeletal muscle dysfunction, depression, gastrointestinal diseases, anemia, and diabetes, and it has been recognized that the presence of some comorbidities could substantially affect the severity and prognosis of the disease.^[3,4]^ Hospitalization for COPD is associated with high mortality especially for intensive care unit (ICU)-admitted patients. While studies have shed light on risk factors for mortality in hospitalized COPD patients including cardiac insufficiency, advanced age, comorbidities, and nutritional status, as well as arterial oxygen and carbon dioxide partial pressures on admission,^[5–8]^ the unique challenges and complexities faced by critically ill patients with COPD necessitate a more focused investigation into the factors that impact outcomes in this particular population.

Acid-base imbalances are often observed in advanced COPD due to respiratory failure contributing to carbon dioxide accumulation leading to respiratory acidosis and hypoxemia, which contributes to reduction of oxygen deliver to major organs leading to anaerobic metabolism and overproduction of lactate, and multiple organ dysfunction, such as renal insufficiency and liver dysfunction, which reduce the elimination of acidic metabolic products, thus leading to metabolic acidosis.^[9–11]^ Once critically ill patients experience an acid-base imbalance, it is difficult to restore balance through treatment.^[12][^Anion gap (AG) is a laboratory parameter that compares the total concentration of blood sodium with chloride and bicarbonate concentrations, usually used to assess acid base disorders and categorize metabolic acidosis.^[13]^ Elevated AG is reported to be related to poor prognosis in critical ill patients, such as sepsis, acute myocardial infarction, acute kidney injury, and infective endocarditis, et al.^[14–17]^ Hyponatremia is an independent risk factor for prognosis of COPD patients and a study from China found that elevated AG is independently associated with hyponatremia in COPD patients.^[18]^ COPD, as a chronic disease, continues to affect patients’ health even after discharge, and the relationship between AG and long-term mortality in critically ill COPD patients remains unexplored.

The study aims to investigate the relationship between AG and 365-day all-cause mortality in critically ill patients with COPD and to look for potential saturation effects that could provide valuable insights into the impact of acid–base imbalance on the prognosis of these susceptible patients.

## 2. Method

### 2.1. Data source

This study is a retrospective analysis based on the Medical Information Mart for Intensive Care-IV database, which contains comprehensive data on over 730,141 patients admitted to ICU at Beth Israel Deaconess Medical Center between 2008 and 2019. Upon completion of the course on the official website, we can download the database for free. One of the authors, Xiaohan Xiu, has obtained a certificate (Record ID: 13205753). The establishment and use of this database have been approved by the review committees of the Massachusetts Institute of Technology and the Beth Israel Deaconess Medical Center. As all data have been de-identified, informed consent is not required.

### 2.2. Study population

**Figure 1.**
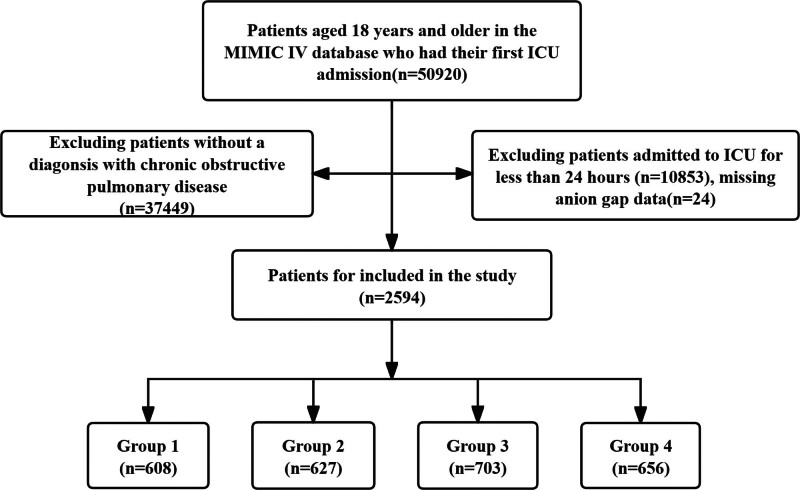
Flow chart of the study subjects.

The study eventually included a total of 2594 participants. The inclusion criteria were: (1) patients aged over 18 years who were admitted to the ICU for the first time; (2) patients with an ICU stay longer than 24 hours; (3) patients diagnosed with COPD based on ICD-9 or ICD-10 codes; and (4) patients with AG values within 24 hours of ICU admission. In the end, a total of 2594 patients were included in the study and divided into 4 groups based on quartiles of AG values (Fig. 1).

### 2.3. Data extraction

Data extraction for this study was conducted using pgAdmin4 (version 7.8). Demographic data of the patients including age and gender were obtained. Additionally, vital signs were measured immediately after ICU hospitalization, including weight, respiratory rate, heart rate, systolic blood pressure (SBP), and diastolic blood pressure (DBP). Comorbidities of COPD were recorded, such as hypertension, type 2 diabetes mellitus (DM), heart failure, myocardial infarction, chronic kidney disease (CKD), stroke, and hyperlipidemia. Laboratory parameters were extracted on the first day of the patient’s ICU admission, including blood AG, white blood cell (WBC), red blood cell (RBC), platelet, hemoglobin, red blood cell volume distribution width (RDW), hematocrit, sodium, potassium, calcium, glucose, prothrombin time (PT), partial thromboplastin time (PTT), urea nitrogen, and creatinine. Sequential organ failure assessment score (SOFA), simplified acute physiology score (SAPS II), acute physiology score (APS III), and systemic inflammatory response syndrome score (SIRS) were calculated upon ICU admission.

The primary outcome of this study was a 365-day mortality.

### 2.4. Statistical analysis

For normally distributed continuous variables, analyze using a *t* test or one-way analysis of variance and express as mean ± standard deviation. For non-normally distributed continuous variables, analyze using the Wilcoxon rank-sum test and express as median and interquartile range [M (Q1, Q3)]. For categorical variables, analyze using the chi-square test and express as frequency and percentage (%).

Univariate and multivariate Cox proportional-hazards models were conducted to evaluate the relationship between AG and 365-day mortality. Four different models were developed based on this: crude model included only AG, Model 1 adjusted for age, sex, and weight, while Model 2 additionally adjusted for WBC, RBC, platelet, hemoglobin, and RDW, and Model 3 further adjusted for hypertension, type 2 DM, heart failure, myocardial infarction, CKD, sodium, potassium, calcium, glucose, PT, PTT, urea nitrogen, creatinine, SOFA, APS III, SIRS, SAPS II, SBP, DBP, heart rate, and respiratory rate. In all 4 models, when AG was considered as a categorical variable, the lowest quartile was used as the reference category. The participants were grouped according to quartiles of AG, and the occurrence rate of the primary outcome was determined using Kaplan–Meier curves. AG was analyzed as a continuous variable using restricted cubic splines to elucidate the dose–response relationship and the correlation of AG with the risk of primary outcome events. If a non-linear relationship was identified, the inflection point of AG was calculated. Additionally, COPD patients were divided into different subgroups based on age, sex, weight, hypertension, type 2 DM, heart failure, myocardial infarction, and CKD. Subgroup analysis and interaction tests were conducted to investigate the associations of these variables across different population subgroups.

All analysis were carried out using R software (version 4.1.3), and *P* < .05 was considered statistically significant.

## 3. Results

### 3.1. Baseline characteristics

Our study included a total of 2594 COPD patients, with an average age of 72.00 years and 47.61% males. AG values ranged from 5.67 to 48.8 mmol/L (median = 14; interquartile range 12–16.33, mmol/L). The study population was divided into 4 groups based on the quartiles of AG at admission (Q1: <12.00, Q2: 12.00–14.00, Q3: 14.00–16.33, and Q4: >16.33, mmol/L). The baseline characteristics of the 4 groups are shown in Table 1. There were statistically significant differences among the 4 groups in age, weight, levels of blood WBC, RBC, platelet, hemoglobin, RDW, hematocrit, sodium, potassium, calcium, glucose, PT, PTT, urea nitrogen and creatinine, SOFA, APSIII, SIRS, SAPS II, SBP, heart rate, respiratory rate, incidence of hypertension, type 2 DM, heart failure, myocardial infarction, CKD and stroke, 90-day and 365-day all-cause mortality (*P* < .05). Patients in the highest quartile of AG were older and had higher WBC, platelet, RDW, calcium, glucose, urea nitrogen, creatinine, heart rate, and respiratory rate. They were also more likely to have CKD, myocardial infarction, heart failure, stroke, and type 2 DM. The 365-day all-cause mortality was higher in the Q4 group compared to the other 3 groups (*P* < .05).

**Table 1:** Characteristics of the study population by AG quartiles.

	Total (n = 2594)	AG quartiles (mmol/L)				*P*-value
		Q1 (n = 608)	Q2 (n = 627)	Q3 (n = 703)	Q4 (n = 656)	
Age, years	72.00 (64.00, 80.00)	71.00 (64.00, 78.00)	72.00 (64.00, 80.50)	72.00 (65.00, 80.00)	73.00 (64.00, 81.00)	.023
Sex, n (%)						.100
Female	1359 (52.39)	336 (55.26)	309 (49.28)	357 (50.78)	357 (54.42)	
Male	1235 (47.61)	272 (44.74)	318 (50.72)	346 (49.22)	299 (45.58)	
Weight, kg	78.32 (65.00, 95.00)	77.10 (64.40, 95.85)	75.00 (63.20, 92.00)	81.80 (66.05, 98.65)	79.10 (65.38, 94.10)	<.001
WBC, K/µL	11.50 (8.40, 15.29)	11.18 (8.25, 14.66)	11.00 (7.90, 14.47)	11.15 (8.36, 15.13)	12.84 (9.12, 17.16)	<.001
RBC, m/µL	3.49 (3.04, 4.01)	3.39 (3.02, 3.82)	3.51 (3.07, 4.03)	3.59 (3.11, 4.08)	3.48 (2.99, 4.03)	<.001
Platelet, K/µL	189.50 (140.62, 253.00)	180.17 (135.75, 231.75)	183.50 (142.14, 246.75)	195.00 (142.75, 256.50)	201.83 (141.15, 267.17)	.008
Hemoglobin, g/dL	10.38 (8.90, 11.80)	10.00 (8.90, 11.35)	10.43 (9.05, 11.85)	10.60 (9.10, 12.18)	10.37 (8.67, 11.80)	<.001
RDW, %	14.75 (13.70, 16.30)	14.57 (13.52, 15.79)	14.70 (13.62, 16.02)	14.65 (13.63, 16.24)	15.19 (14.00, 16.80)	<.001
Hematocrit, %	32.12 (28.00, 36.45)	31.19 (27.80, 35.08)	32.30 (28.12, 36.80)	32.80 (28.60, 37.35)	32.28 (27.30, 36.40)	<.001
Sodium, mmol/L	139.00 (136.00, 141.33)	139.00 (137.00, 141.50)	139.00 (136.00, 141.33)	139.00 (136.00, 141.00)	138.00 (135.00, 141.00)	<.001
Potassium, mmol/L	4.28 (3.95, 4.68)	4.35 (4.03, 4.70)	4.23 (3.97, 4.60)	4.20 (3.90, 4.60)	4.30 (3.90, 4.83)	<.001
Calcium, mg/dL	8.40 (7.98, 8.80)	8.30 (7.95, 8.70)	8.40 (8.00, 8.80)	8.45 (8.00, 8.85)	8.50 (7.96, 8.90)	<.001
Glucose, mg/dL	130.00 (110.00, 163.00)	122.50 (107.00, 144.50)	128.00 (106.16, 153.50)	133.50 (111.50, 168.33)	145.00 (116.00, 188.00)	<.001
PT, s	13.90 (12.40, 16.20)	13.90 (12.69, 15.38)	13.65 (12.30, 15.45)	14.02 (12.30, 16.38)	14.10 (12.40, 17.66)	.003
PTT, s	32.62 (28.10, 42.83)	31.19 (27.95, 39.10)	32.00 (27.90, 40.71)	33.00 (28.04, 43.69)	34.30 (28.70, 47.53)	<.001
Urea nitrogen, mg/dL	21.00 (14.75, 34.00)	17.00 (13.00, 23.75)	19.00 (13.50, 28.00)	22.75 (15.50, 34.50)	30.59 (18.50, 53.00)	<.001
Creatinine, mg/dL	1.00 (0.70, 1.48)	0.80 (0.63, 1.05)	0.90 (0.70, 1.20)	1.05 (0.78, 1.53)	1.47 (0.95, 2.60)	<.001
SOFA	4.00 (2.00, 7.00)	4.00 (2.00, 6.00)	4.00 (2.00, 6.00)	4.00 (2.00, 7.00)	6.00 (3.00, 9.00)	<.001
APS III	42.00 (32.00, 55.00)	37.00 (29.00, 48.00)	39.00 (30.00, 50.00)	42.00 (32.00, 54.00)	52.00 (39.00, 65.00)	<.001
SAPS II	37.00 (30.00, 45.00)	35.00 (28.00, 42.00)	35.00 (29.00, 43.00)	37.00 (30.00, 44.50)	41.00 (33.00, 52.00)	<.001
SIRS	3.00 (2.00, 3.00)	3.00 (2.00, 3.00)	3.00 (2.00, 3.00)	3.00 (2.00, 3.00)	3.00 (2.00, 3.00)	<.001
Heart rate, beats per minute	84.68 (75.34, 96.02)	83.06 (75.60, 92.72)	83.73 (73.20, 93.09)	83.88 (74.77, 95.81)	89.13 (77.28, 99.91)	<.001
Respiratory rate, breaths per minute	19.65 (17.44, 22.19)	18.74 (16.83, 21.36)	19.52 (17.35, 21.77)	19.79 (17.77, 22.26)	20.43 (18.08, 23.24)	<.001
SBP, mm Hg	114.58 (106.14, 126.21)	112.88 (104.99, 123.37)	115.04 (107.00, 126.80)	116.56 (107.25, 128.75)	114.41 (104.84, 125.85)	<.001
DBP, mm Hg	63.19 (57.07, 70.00)	62.50 (57.05, 69.11)	63.00 (57.00, 70.16)	63.82 (57.18, 70.46)	63.57 (57.25, 70.05)	.445
Hypertension, n (%)						<.001
No	1554 (59.91)	342 (56.25)	362 (57.74)	409 (58.18)	441 (67.23)	
Yes	1040 (40.09)	266 (43.75)	265 (42.26)	294 (41.82)	215 (32.77)	
Type 2 DM, n (%)						<.001
No	1734 (66.85)	429 (70.56)	460 (73.37)	453 (64.44)	392 (59.76)	
Yes	860 (33.15)	179 (29.44)	167 (26.63)	250 (35.56)	264 (40.24)	
Heart failure, n (%)						<.001
No	1460 (56.28)	384 (63.16)	343 (54.70)	392 (55.76)	341 (51.98)	
Yes	1134 (43.72)	224 (36.84)	284 (45.30)	311 (44.24)	315 (48.02)	
Myocardial infarction, n (%)						<.001
No	2260 (87.12)	551 (90.62)	571 (91.07)	607 (86.34)	531 (80.95)	
Yes	334 (12.88)	57 (9.38)	56 (8.93)	96 (13.66)	125 (19.05)	
CKD, n (%)						<.001
No	1977 (76.21)	532 (87.50)	504 (80.38)	517 (73.54)	424 (64.63)	
Yes	617 (23.79)	76 (12.50)	123 (19.62)	186 (26.46)	232 (35.37)	
Stroke, n (%)						.005
No	2361 (91.02)	569 (93.59)	580 (92.50)	631 (89.76)	581 (88.57)	
Yes	233 (8.98)	39 (6.41)	47 (7.50)	72 (10.24)	75 (11.43)	
Hyperlipidemia, n (%)						.596
No	1402 (54.05)	317 (52.14)	335 (53.43)	384 (54.62)	366 (55.79)	
Yes	1192 (45.95)	291 (47.86)	292 (46.57)	319 (45.38)	290 (44.21)	
90-day mortality, n (%)						<.001
No	1938 (74.71)	516 (84.87)	489 (77.99)	527 (74.96)	406 (61.89)	
Yes	656 (25.29)	92 (15.13)	138 (22.01)	176 (25.04)	250 (38.11)	
365-day mortality, n (%)						<.001
No	1654 (63.76)	457 (75.16)	414 (66.03)	445 (63.30)	338 (51.52)	
Yes	940 (36.24)	151 (24.84)	213 (33.97)	258 (36.70)	318 (48.48)	

All values are expressed as a proportion (%) or mean ± standard deviation.

APS III = acute physiology score III, CKD = chronic kidney disease, DBP = diastolic blood pressure, PT = prothrombin time, PTT = partial thromboplastin time, RBC = red blood cell, RDW = red blood cell distribution width, SAPS II = simplified acute physiology score, SBP = systolic blood pressure, SIRS = systemic inflammatory response syndrome, SOFA = sequential organ failure assessment, WBC = white blood cell.

Table 2 classified patients into survivor and non-survivor groups based on whether they survived or died within 365 days of ICU admission. There were 940 cases in the non-survivor group and 1654 cases in the survivor group. Compared to survivors, patients in the non-survivor group were older and had lower weight. In the non-survivor group, levels of WBC, RDW, potassium, glucose, urea nitrogen, creatinine, PT, PTT, and AG were significantly higher, while heart rate and respiratory rate were significantly increased. Additionally, RBC, hemoglobin, hematocrit, SBP, DBP, heart rate, and respiratory rate were significantly lower in the non-survivor group compared to the survivor group. Non-survivors tended to have a more severe clinical presentation, as evidenced by significantly higher SAPS II, SOFA, APS III, and SIRS. In terms of comorbidities, the non-survivor group had significantly more cases of CKD, myocardial infarction, and heart failure, while the number of individuals with hypertension and hyperlipidemia was significantly lower in the non-survivor group compared to the survivor group.

**Table 2:** Characteristics of the study population according to whether died within 365 days.

	Total (n = 2594)	Survivor (n = 1654)	Non-survivor (n = 940)	*P*-value
Age, years	71.75 (60.67, 82.83)	70.129 (59.3, 80.94)	74.61 (63.65, 85.57)	<.001
Sex, n (%)				.711
Female	1359 (52.39)	862 (52.12)	497 (52.87)	
Male	1235 (47.61)	792 (47.88)	443 (47.13)	
Weight, kg	81.79 (56.96, 106.62)	83.83 (58.64, 109.02)	78.18 (54.4101.96)	<.001
WBC, K/µL	12.92 (2.86, 10.06)	12.53 (4.25, 20.81)	13.59 (1.03, 26.15)	.021
RBC, m/µL	3.55 (2.85, 4.25)	3.62 (2.93, 4.31)	3.41 (2.71, 4.11)	<.001
Platelet, K/µL	206.01 (109.27, 302.75)	206.08 (114.08, 298.08)	205.89 (101.28, 310.5)	.963
Hemoglobin, g/dL	10.52 (8.47, 12.57)	10.77 (8.72, 12.82)	10.07 (8.11, 12.03)	<.001
RDW, %	15.29 (12.96, 17.62)	14.89 (12.77, 17.01)	16 (13.48, 18.52)	<.001
Hematocrit, %	32.69 (26.51, 38.87)	33.32 (27.14, 39.5)	31.57 (25.56, 37.58)	<.001
Sodium, mmol/L	138.45 (133.59, 143.31)	138.42 (133.93, 142.91)	138.49 (133.03, 143.95)	.726
Potassium, mmol/L	4.34 (3.75, 4.93)	4.31 (3.75, 4.87)	4.39 (3.76, 5.02)	.002
Calcium, mg/dL	8.4 (7.72, 9.08)	8.39 (7.76, 9.02)	8.41 (7.67, 9.15)	.592
Glucose, mg/dL	144.51 (88.62, 200.4)	141.93 (88.26, 195.6)	149.03 (89.67, 208.39)	.002
PT, s	15.69 (8.5, 22.88)	15.03 (8.68, 21.38)	16.85 (8.51, 25.19)	<.001
PTT, s	39.18 (20.4, 57.96)	37.66 (19.93, 55.39)	41.87 (21.65, 62.09)	<.001
Urea nitrogen, mg/dL	28 (6.83, 49.17)	24.01 (5.62, 42.4)	35.01 (11.24, 58.78)	<.001
Creatinine, mg/dL	1.35 (0.16, 2.54)	1.2 (0.18, 2.22)	1.62 (0.21, 3.03)	<.001
SOFA	5.01 (1.55, 8.47)	4.39 (1.33, 7.45)	6.1 (2.27, 9.93)	<.001
APS III	45.41 (26.39, 64.43)	40.92 (24.98, 56.86)	53.32 (32.02, 74.62)	<.001
SAPS II	38.72 (25.87, 51.57)	35.78 (24.54, 47.02)	43.91 (30.07, 57.75)	<.001
SIRS	2.56 (1.68, 3.44)	2.51 (1.63, 3.39)	2.66 (1.8, 3.52)	<.001
Heart rate, beats per minute	86.08 (70.95, 101.21)	84.88 (70.52, 99.24)	88.18 (71.99, 104.37)	<.001
Respiratory rate, breaths per minute	19.98 (16.34, 23.62)	19.54 (16.06, 23.02)	20.76 (16.97, 24.55)	<.001
SBP, mm Hg	116.9 (101.09, 132.71)	117.73 (101.89, 133.57)	115.45 (99.79, 131.11)	<.001
DBP, mm Hg	64.1 (53.62, 74.58)	64.81 (54.4, 75.22)	62.85 (52.35, 73.35)	<.001
AG, mmol/L	14.33 (10.7, 17.96)	13.84 (10.43, 17.25)	15.2 (11.37, 19.03)	<.001
Hypertension, n (%)				<.001
No	1554 (59.91)	921 (55.68)	633 (67.34)	
Yes	1040 (40.09)	733 (44.32)	307 (32.66)	
Type 2 DM, n (%)				.838
No	1734 (66.85)	1108 (66.99)	626 (66.60)	
Yes	860 (33.15)	546 (33.01)	314 (33.40)	
Heart failure, n (%)				<.001
No	1460 (56.28)	1018 (61.55)	442 (47.02)	
Yes	1134 (43.72)	636 (38.45)	498 (52.98)	
Myocardial infarction, n (%)				.002
No	2260 (87.12)	1467 (88.69)	793 (84.36)	
Yes	334 (12.88)	187 (11.31)	147 (15.64)	
CKD, n (%)				<.001
No	1977 (76.21)	1336 (80.77)	641 (68.19)	
Yes	617 (23.79)	318 (19.23)	299 (31.81)	
Stroke, n (%)				.172
No	2361 (91.02)	1515 (91.60)	846 (90.00)	
Yes	233 (8.98)	139 (8.40)	94 (10.00)	
Hyperlipidemia, n (%)				.001
No	1402 (54.05)	854 (51.63)	548 (58.30)	
Yes	1192 (45.95)	800 (48.37)	392 (41.70)	

All values are expressed as a proportion (%) or mean ± standard deviation.

AG = anion gap, APS III = acute physiology score III, CKD = chronic kidney disease, DBP = diastolic blood pressure, PT = prothrombin time, PTT = partial thromboplastin time, RBC = red blood cell, RDW = red blood cell distribution width, SAPS II = simplified acute physiology, SBP = systolic blood pressure, SIRS = systemic inflammatory response syndrome, SOFA = sequential organ failure assessment, WBC = white blood cell.

### 3.2. Relationship between AG and 365-day all-cause mortality in COPD critically ill patients

**Figure 2.**
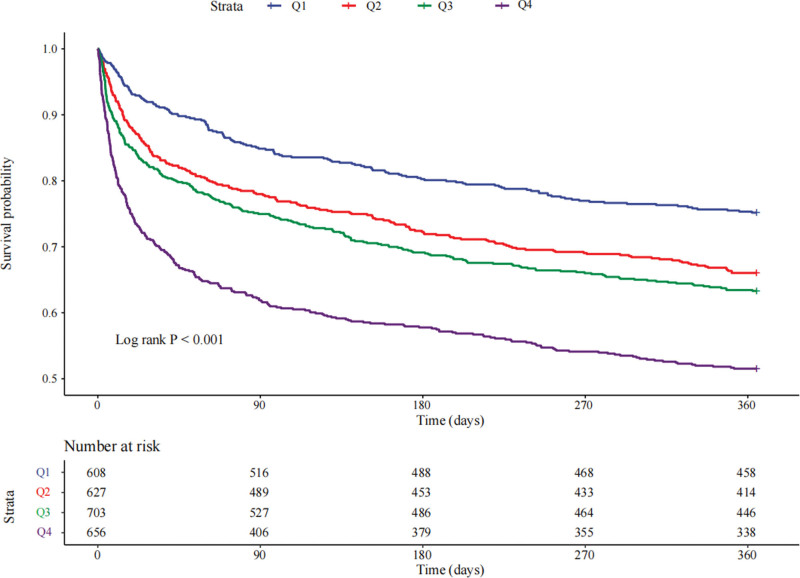
The Kaplan–Meier (KM) survival curves are classified by AG quartiles. AG = anion gap.

Kaplan–Meier curves showed that the prevalence of 365-day all-cause mortality among the 4 groups were different (Fig. 2). Compared to patients with lower AG levels, those with the highest AG level had the lowest 365-day survival probability (log-rank *P* < .001).

To investigate the independent impact of AG on the 365-day all-cause mortality in COPD critically ill patients, 4 Cox models were applied (Table 3). When AG was treated as a continuous variable, the crude model revealed a significant association between AG and the mortality of COPD patients (hazard ratio [HR] = 1.09, 95% confidence interval [CI]: 1.07–1.10). After adjusting for confounding factors, Model 3 still showed a significant association between AG and the mortality of critically ill patients with COPD (HR = 1.03, 95% CI: 1.01–1.05). When AG was treated as a categorical variable using quartiles, in Model 3 adjusting for relevant variables, the adjusted HR (95% CI) from lowest to highest AG were as follows: 1.00, 1.34 (1.09–1.66), 1.44 (1.17–1.78), and 1.49 (1.19–1.87).

**Table 3:** Association between AG and 365-day all-cause mortality in critically ill patients with COPD.

	Crude model		Model 1		Model 2		Model 3	
	HR (95% CI)	*P*-value	HR (95% CI)	*P*-value	HR (95% CI)	*P*-value	HR (95% CI)	*P*-value
AG	1.09 (1.07, 1.11)	<.001	1.09 (1.07, 1.11)	<.001	1.08 (1.06, 1.10)	<.001	1.03 (1.01, 1.05)	.005
AG quartiles								
Q1	Ref		Ref		Ref		Ref	
Q2	1.47 (1.19, 1.81)	<.001	1.39 (1.13, 1.71)	.002	1.43 (1.16, 1.76)	<.001	1.34 (1.09, 1.66)	.010
Q3	1.64 (1.34, 2.00)	<.001	1.60 (1.30, 1.95)	<.001	1.60 (1.31, 1.96)	<.001	1.44 (1.17, 1.78)	<.001
Q4	2.47 (2.03, 2.99)	<.001	2.37 (1.96, 2.88)	<.001	2.27 (1.86, 2.76)	<.001	1.49 (1.19, 1.87)	<.001
*P* for trend		<.001		<.001		<.001		<.001

Crude model: no adjusted.

Model 1: adjusted for age, sex, and weight.

Model 2: adjusted for age, sex, weight, WBC, RBC, Platelet, hemoglobin, and RDW.

Model 3: adjusted for age, sex, weight, WBC, RBC, platelet, hemoglobin, RDW, hypertension, type 2 DM, heart failure, myocardial infarction, CKD, sodium, potassium, calcium, glucose, PT, PTT, urea nitrogen, creatinine, SOFA, APS III, SIRS, SAPS II, SBP, DBP, heart rate, and respiratory rate.

APS III = acute physiology score III, CKD = chronic kidney disease, DBP = diastolic blood pressure, PT = prothrombin time, PTT = partial thromboplastin time, RBC = red blood cell, RDW = red blood cell distribution width, SAPS II = simplified acute physiology, SBP = systolic blood pressure, SIRS = systemic inflammatory response syndrome, SOFA = sequential organ failure assessment, WBC = white blood cell.

### 3.3. Nonlinearity relationship

**Figure 3.**
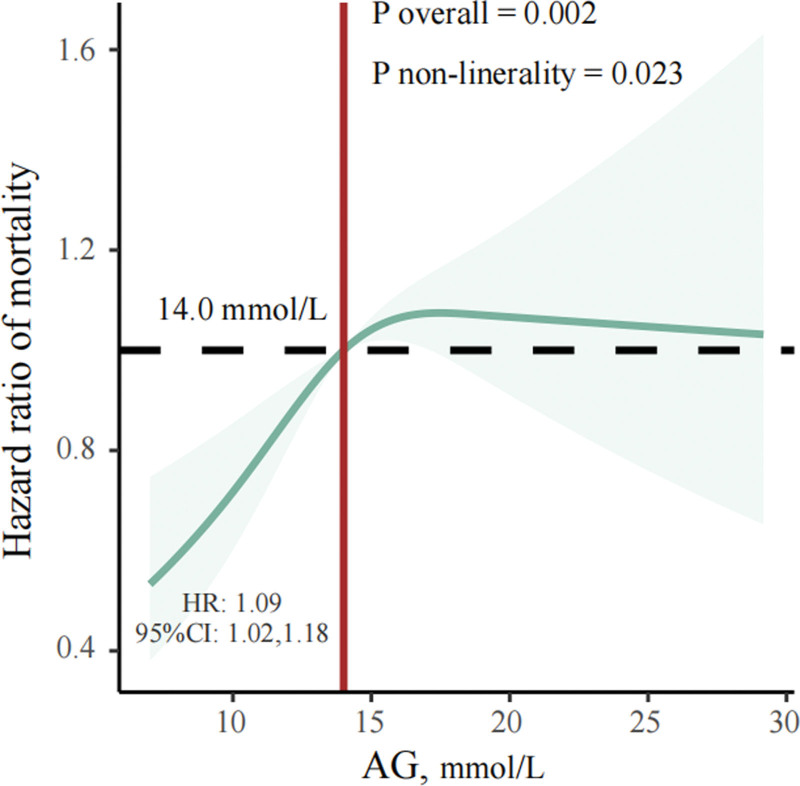
Restricted cubic spline (RCS) regression analysis of AG with 365-day all-cause mortality. AG = anion gap.

Restricted cubic spline curve was used to examine the non-linear relationship between AG and the 365-day all-cause mortality (Fig. 3). After adjusting for confounding factors, the association between AG and all-cause mortality of critically ill patients with COPD remained statistically significant (overall *P* = .002). A nonlinear association was also shown between AG and COPD critically ill patients (non-linear *P* = .023). That is, 365-day all-cause mortality in COPD critically ill patients increases with increasing AG, and after a saturation is reached, the risk of death no longer rises. We went a step further and determined the saturation for AG to be 14.00 mmol/L. Finally, we found that the specific HR and its 95% CI were 1.09 (1.02–1.18) when the AG value was <14.00 mmol/L.

### 3.4. Subgroup analysis

**Figure 4.**
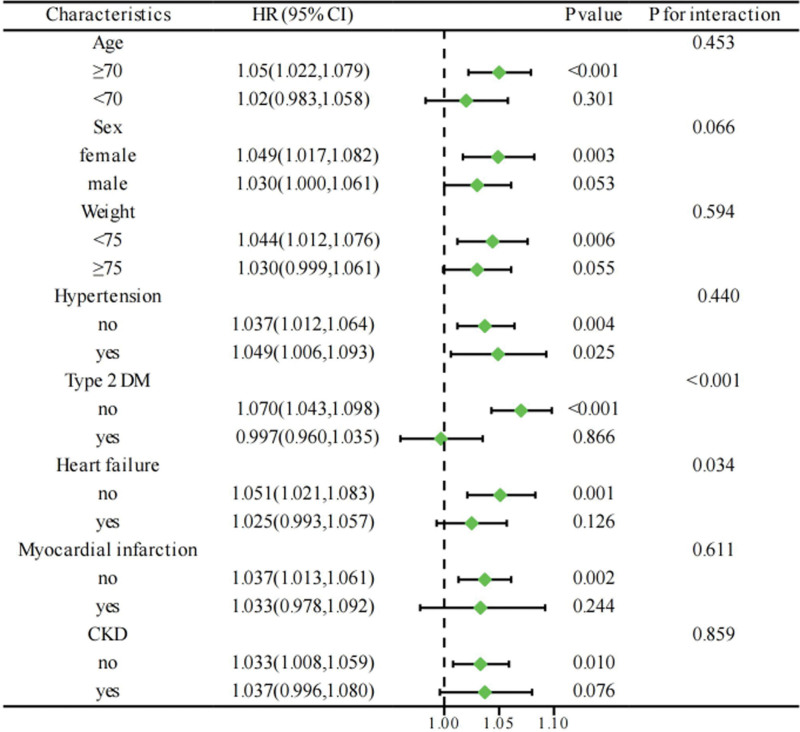
Subgroup analysis of the association between AG and 365-day all-cause in COPD critical ill patients. AG = anion gap, COPD = chronic obstructive pulmonary disease.

COPD patients were divided into different subgroups based on age, sex, weight, hypertension, type 2 DM, heart failure, myocardial infarction, and CKD. The impact of AG on the 365-day all-cause mortality of COPD critical ill patients was studied and the results were presented in a forest plot (Fig. 4). The results showed that the association between AG and all-cause mortality was positive in most subgroups. Further interactive analysis revealed a statistically significant difference in the relationship between AG and COPD patient mortality in the type 2 DM subgroup, indicating an impact of type 2 DM on the relationship between AG and COPD patient mortality (*P* for interaction < .05).

## 4. Discussion

To the best of our knowledge, this is the first investigation of the association between AG and long-term mortality in critically ill patients with COPD and exposes a saturation effect of AG. Multivariate analysis showed that AG was an independent risk factor for 365-day mortality in COPD critically ill patients, and high AG was associated with higher 365-day all-cause mortality (HR 1.49; 95% CI 1.19–1.87; *P* < .001). Subgroup analysis further confirmed the stable relationship between AG and 365-day mortality.

AG is recognized as a prognostic biomarker for patients hospitalized in intensive care units and elevated AG is strongly associated with mortality in sepsis^[19]^ and various cardiovascular and cerebrovascular diseases including acute myocardial infarction, heart failure,^[20]^ cerebral infarction.^[20–22]^ The kidneys play an important role in the regulation of acid-base balance. With progressive loss of kidney function, acid-base disturbance inevitably occurs. Asahina et al. found that among patients with advanced CKD, high AG was associated with a higher rate of progression to kidney failure requiring replacement therapy and death.^[23]^ A study including 11,957 noninstitutionalized civilian population from across the United States found that increase of AG occurred in early kidney disease, and were associated with high risk of mortality independent of eGFR.^[^^24^^]^ Similarly, in respiratory diseases, elevated AG was found to be associated with increased 30-day mortality in patients with severe asthma.^[25]^ In keeping with these findings, our study demonstrated that high AG levels were strongly associated with long-term mortality in critically ill patients with COPD, and this association remained significant even after adjusting for confounders.

Although no study has elaborated on the mechanistic role of AG in the prognosis of critically ill patients with COPD, from the studies published so far, we hypothesize that lactate-induced metabolic acidosis may play an intermediate role. High AG values may indicate excess acid buildup such as lactic acid, and lactic acidosis is one of the most common causes of metabolic acidosis in critically ill patients because of reduced tissue oxygenation due to severe hypoxemia or reduced perfusion.^[12]^ Elevated lactate levels have been confirmed by several studies to be associated with increased mortality.^[26,27]^ These suggest that the mechanism of positive relationship between AG and mortality in patients with COPD may be lactic acid accumulation due to hypoxia and inadequate tissue perfusion in critical ill patients.

Subgroup analysis showed differences in the subgroups with type 2 DM, which may be due to decreased insulin, increased counter-regulatory hormones, and increased ketone bodies due to lipolysis in patients with type 2 DM, which ultimately leads to diabetic ketoacidosis.^[12]^ This effect may influence the effect of the acid–base marker AG on all-cause mortality.

Our study has some limitations. First, although this was a large cohort study involving more than 2000 patients, it could not avoid the bias inherent in retrospective studies. Second, the study did not include an intervention for outcome, and the lack of these data may have affected the results of the analysis. In addition, the MIMIC database is based on patients hospitalized over the past 20 years, so further validation of the applicability of our findings to current clinical practice is needed. Future studies should include animal experiments and other prospective studies to develop additional predictors for patients with severe COPD.

## 5. Conclusion

In summary, our study suggests that AG is an independent risk factor for long-term mortality in critically ill patients with COPD and that AG has a saturation effect on mortality. which may provide valuable insights into the post-discharge management to improve long-term prognosis of critically ill patients with COPD.

## Acknowledgments

We gratefully acknowledge the MIMIC-IV database, clinical staff, and participants for their contributions to this study.

## Author contributions

**Conceptualization:** Xiaohan Xiu, Xuemei Chen.

**Data curation:** Xiaohan Xiu, Zhenyu Yang.

**Formal analysis:** Zhenyu Yang, Xuemei Chen.

**Funding acquisition:** Xuemei Chen.

**Investigation:** Xiaohan Xiu, Zhenyu Yang.

**Methodology:** Xiaohan Xiu, Zhenyu Yang, Xuemei Chen.

**Project administration:** Xuemei Chen.

**Resources:** Xiaohan Xiu, Zhenyu Yang.

**Software:** Xiaohan Xiu, Zhenyu Yang.

**Supervision:** Xiaohan Xiu.

**Validation:** Xiaohan Xiu, Zhenyu Yang.

**Visualization:** Xiaohan Xiu.

**Writing – original draft:** Xiaohan Xiu, Zhenyu Yang.

**Writing – review & editing:** Xuemei Chen.
